# Effect of siRNA-Livin on drug resistance to chemotherapy in glioma U251 cells and CD133^+^ stem cells

**DOI:** 10.3892/etm.2015.2675

**Published:** 2015-08-10

**Authors:** YANG LIU, QIANG GUO, HAO ZHANG, GEN-HUA LI, SONG FENG, XI-ZHEN YU, LING-SHENG KONG, LEI ZHAO, FENG JIN

**Affiliations:** 1 Graduate School, Tianjin Medical University, Tianjin 300070, P.R. China; 2 Department of Neurosurgery, Neuro-oncology Laboratory, Affiliated Hospital of Jining Medical College, Jining, Shandong 272029, P.R. China; 3 Department of Hepatology & Infectious Disease, Union Hospital, Tongji Medical College, Huazhong University of Science and Technology, Wuhan, Hubei 430022, P.R. China

**Keywords:** glioblastoma, cancer stem cell, Livin, multidrug resistance-associated protein, drug resistance

## Abstract

The aim of the present study was to observe the effect of siRNA-Livin on the expression of multidrug resistance-associated protein (MRP) genes in a U251 cell line and U251 stem cells. CD133^+^ cancer stem cells were identified and isolated from the U251 glioblastoma cells, and morphological observations were used to detect the cell survival conditions. In addition, quantitative polymerase chain reaction was used to detect the mRNA expression levels of Livin, MRP1 and MRP3. Following transfection with the lentivirus containing the siRNA-Livin, the expression of Livin was significantly inhibited in the U251 cells and stem cells (P<0.01). Following temozolomide intervention, the proliferation of the U251 cells and U251 stem cells was restrained, with a lot of cell debris present and the structure of the cell spheres destroyed. The inhibitory effect was more significant following transfection with siRNA-Livin. Prior to siRNA-Livin transfection, the expression of MRP1 presented an increasing trend in the U251 cells and U251 stem cells with increasing drug concentrations and intervention times (P<0.05). Following siRNA-Livin transfection, the expression of MRP1 decreased in the U251 cells and U251 stem cells under the same drug concentration and intervention time (P<0.05), while the expression of MRP3 increased in the U251 stem cells under the same intervention concentration and time (P<0.05). Therefore, siRNA-Livin was shown to decrease the expression of MRP1 in U251 cells and U251 stem cells, increase the expression of MRP3 in U251 stem cells and decrease the proliferation of U251 cells and U251 stem cells. Thus, Livin may be associated with the high expression of MRP1, and siRNA-Livin may be used to lower the expression of MRP1 in order to reduce the drug resistance to chemotherapy in cases of glioblastoma.

## Introduction

Glioblastoma multiforme is the most common and severe type of brain tumor, which presents unique challenges to therapy due to its location, aggressive biological behavior and diffuse infiltrative growth. The survival times of patients with a glioblastoma are very short. Even with combined treatment including complete surgery, radiotherapy and chemotherapy, the survival time is estimated to be between 12 and 18 months following diagnosis (1). Chemotherapy is often used as a secondary treatment method for glioblastomas following the removal of the tumor by surgery, in order to prevent tumor recurrence (2). Since a curative outcome is unable to be achieved by surgery only, chemotherapy has become an essential adjuvant therapy for glioblastomas following surgery. Thus, the key to successfully achieving remission in glioma cases is to target the remaining tumor cells, including glioma stem cells (GSCs), by thorough chemotherapy following surgery.

The existence of drug resistance to chemotherapy in glioma cases has led to the inefficiency of chemotherapeutic drugs and the increased risk of tumor recurrence following treatment (3). In recent years, the establishment of the ‘cancer stem cell theory’ and the further study of multidrug resistance-associated protein (MRP)1 and 3 genes have provided a new research direction for glioma chemotherapy drug resistance. Compared with normal glioma cells, a stronger drug resistance to chemotherapy can be observed in the stem cells isolated from gliomas (4). The main reason underlying the chemotherapy drug resistance of glioma is the ability of GSCs to generate strong chemotherapeutic drug resistance, resulting in the patient becoming resistant to chemotherapy drugs and ultimately leading to tumor recurrence (5). According to the cancer stem cell hypothesis, the key to completely removing cancer cells is not only targeting the remaining glioma cells, but also targeting the cancer stem cells. Thus, GSCs have become increasingly studied with regard to chemotherapy drug resistance.

Livin (also known as KIAP or ML-IAP) is a member of the apoptosis protein suppressor (inhibitor of apoptosis protein; IAP) family. Among the eight members of the IAP family, only Livin has two subunits with α and β structures; which are combined to exert a stronger antiapoptotic function compared with the other members of the IAP family (6). Livin has been shown to play a key role in cell apoptosis, cell proliferation and the cell cycle (7). Previous studies demonstrated that Livin was overexpressed in glioblastoma, and that a correlation existed between Livin and chemotherapy drug resistance (8,9). Thus, the present study assessed Livin as a target, and conducted lentiviral transfection of siRNA-Livin in order to investigate the effect of Livin on chemotherapy drug resistance in a glioma U251 cell line and U251 CD133^+^ stem cells.

## Materials and methods

### 

#### Chemicals and reagents

Dulbecco's modified Eagle's medium/nutrient mixture F-12 Ham's (DMEM/F12) with high glucose medium was purchased from GE Healthcare (HyClone; Logan, UT, USA). Fetal bovine serum (FBS), trypsin, streptomycin, benzopenicillin and B-27 (1X) serum-free Supplement were purchased from Gibco Life Technologies (Grand Island, NY, USA). Epidermal growth factor (EGF), basic fibroblast growth factor (bFGF) and leukemia inhibitory factor (LIF) were obtained from Peprotech (Rocky Hill, NJ, USA). A CD133 cell isolation kit (magnetic-activated cell sorting method) was purchased from Miltenyi Biotec GmbH (Bergisch Gladbach, Germany), while the lentivirus was provided by Shanghai GeneChem Co., Ltd. (Shanghai, China). The Cell Counting Kit-8 (CCK-8) was obtained from Dojindo Molecular Technologies, Inc. (Kumamoto, Japan), while temozolomide (TMZ) was purchased from Tasly Pharmaceutical Co., Ltd. (Tianjin, China). SYBR Green I fluorochrome was purchased from Biotium, Inc. (Hayward, CA, USA) and MMLV Reverse Transcriptase was purchased from Aidlab Biotechnologies, Co., Ltd. (Beijing, China).

#### Glioma cell line culture

A glioma U251 cell line was purchased from the China Center for Typical Culture Collection (Wuhan, China). The cell line was cultured in DMEM/F12, containing 10% FBS, 100 µg/ml streptomycin and 100 U/ml benzylpenicillin, under conditions of 37°C, 5% CO2 and saturated humidity.

#### Isolation, identification and cultivation of CD133^+^ GSCs

The isolation and identification of CD133^+^ GSCs was performed according to previous studies (10,11). In brief, the U251 cells were collected and cultivated in serum-free medium [neural stem cell (NSC) medium], containing DMEM/F12 with high glucose medium, 20 ng/ml EGF, 20 ng/ml bFGF, 10 ng/ml LIF and B-27 (1X) Supplement, under conditions of 37°C, 5% CO2 and saturated humidity. The medium was replaced every 3–4 days. After 8–10 days of cultivation, a large number of neurospheres was observed. The neurospheres were collected and the CD133^+^ cells were separated from the spheres using a magnetic-activated cell sorting technique. The sorting processes were performed according to the instructions of the CD133 cell isolation kit. Following cell sorting, the CD133^+^ cells were cultivated in NSC medium.

#### Immunofluorescence staining

Well-grown cell spheres were selected for growing on slides coated with polylysine. After drying at 37°C, the slides were washed with phosphate-buffered saline (PBS) three times. The cells were fixed with paraform for 30 min at room temperature, then washed with PBS a further three times. After blocking with 5% goat serum at 37°C for 30 min, primary monoclonal rabbit anti-human nestin (1:30; BA1289, Boster, Wuhan, China) diluted in 1% BSA was added and the cells were placed in a wet box overnight. Subsequently, the cells were washed with PBS, then secondary monoclonal goat anti-rabbit IgG-fluorescein isothiocyanate antibody (1:50; BA1105, Boster) diluted in 1% BSA was added for incubation for 30 min at 37°C. In addition, a negative control in which PBS was used instead of the primary antibody was performed. The slides were observed using an Olympus BX51 fluorescence microscope (Olympus Corporation, Tokyo, Japan). Immunofluorescent assays for glial fibrillary acidic protein (GFAP) and β-tubulin in the differentiated stem cells were conducted using an identical protocol as that used for nestin, with the exception of the respective antibodies. GFAP and β-tubulin were used to identify glioma cells. They were detected by immunofluorescence using monoclonal GAFP (1:30; BA0056; Boster) antibody diluted in 1% BSA and monoclonal β-tubulin (1:30; BM1453; Boster).

#### Lentiviral transfection

According to a preliminary experiment, the multiplicity of infection was five for the U251 cells and ten for the GSCs. U251 cells were transfected with the lentivirus for 10 h in a six-well plate containing DMEM/F12 with 10% FBS. After 10 h, the medium was replaced and the cells were continually cultured for three days prior to confocal microscopy. CD133^+^ stem cells were transfected with the lentivirus for 20 h in a six-well plate containing the NSC medium. After 20 h, the medium was replaced and the cells were cultured for three days in the NSC medium prior to confocal microscopy. The whole transfection process was performed in a biosafety cabinet. The cells were cultivated under conditions of 37°C, 5% CO2 and saturated humidity.

#### Cell morphology observations and cell proliferation inhibitory rate assessment

Following drug intervention for 24, 48 and 72 h, cell morphology was observed. The cell proliferation inhibitory rate was determined using Cell Counting Kit-8 (CCK-8) solution, according to the manufacturer's instructions. Cells were seeded into a 96-well plate and 10 µl CCK-8 solution was added, after which the plates were incubated for 4 h at 37°C. The absorbance of each well was measured at 450 nm using an automated ELISA reader (Bio-Tek Instruments, Inc., Winookski, VT, USA). Experiments were repeated three times, and the average value was calculated as the inhibition rate. The proliferation inhibitory rate was calculated using the optical density (OD) values as follows: [(Negative control group OD value − experiment Group OD value)/negative control group OD value] × 100%.

#### Quantitative polymerase chain reaction (PCR)

Quantitative PCR was conducted, as previously described (10,11). Cell samples (10^6^) were collected and 1 ml TRIzol reagent was added (Gibco Life Technologies) to obtain the total RNA of the U251 cells and GSCs, according to the manufacturer's instructions. The RNA solution was stored at −80°C until required for further use. All reactions were performed in duplicate, with a negative control that contained no template. The mean value of the threshold cycle (start of exponential amplification) of each sample was normalized against the threshold cycle value of glyceraldehyde-3-phosphate dehydrogenase to obtain the ΔCt value. Quantitative PCR was performed in an ABI-7700 Sequence Detector (Applied Biosystems, Foster City, CA, USA). The initial reverse transcription step was performed using MMLV Reverse Transcriptase. The reverse transcription reaction system included 5.5 µl H2O, 1.0 µl Oligo (dT)18 (50 µg/ml) and 6.0 µl total RNA, which was incubated at 70°C for 5 min and then kept on ice to unfold the secondary structure of the mRNA. Next, 0.5 µl RNasin (40 U/µl), 4.0 µl 5X buffer, 2.0 µl dNTP (10 mM) and 1.0 µl RTase (200 U/µl) was added and the reaction system was heated to 42°C for 60 min and 95°C for 5 min, followed by cooling to 4°C. Quantitative PCR was performed using SYBR Green I fluorochrome. A standard curve was obtained from which the cycle threshold (Ct) value was calculated. Each 50-µl PCR system contained 1/50 of the original cDNA, 7 µl MgCl_2_ (25 mM), 0.8 µl each primer (20 pmol/µl), 1 µl dNTP (10 mM), 1 µl SYBR Green I, 0.5 µl Taq DNA polymerase (5 U/µl; Promega Corporation, Madison, WI, USA) and 5 µl 10X buffer. Following an initial denaturation at 94°C for 3 min, 50 cycles of amplification were performed with a reaction cycle of 94°C for 30 sec, 57°C for 30 sec and 72°C for 30 sec. The fluorescence signal was detected at the end of each cycle. Melting curve analysis was used to confirm the specificity of the products. The 2^−Δ∆Ct^ method was used to calculate the relative expression levels (12). The primers used were as follows: Human-Livin forward, 5′-ACA GAG GAG GAA GAG GAG GAG G-3 and reverse, 5′-GCA GTC AGC GGC CAG TCA TAG-3; human-MRP1 forward, 5′-CAC CAC TGG AGC ATT GAC TACC-3 and reverse, 5′-GTA ATT ACA GCA AGC CTG GAA CC-3′; and human-MRP3 forward, 5′-CCT GTA TGT GGG TCA AAG TGCG-3′ and reverse, 5′-CCC AGC CTC AGG GAA GTG TTG-3.

#### Statistical analysis

Experiments were performed in triplicate and the data are presented as the mean ± standard deviation. Comparisons between data were performed with the Student's t-test, where P<0.05 was considered to indicate a statistically significant difference. Statistical analysis was performed using SPSS 17.0 software (SPSS, Inc., Chicago, IL, USA).

## Results

### 

#### Isolation and differentiation of CD133^+^ stem cells

**Figure 1.**
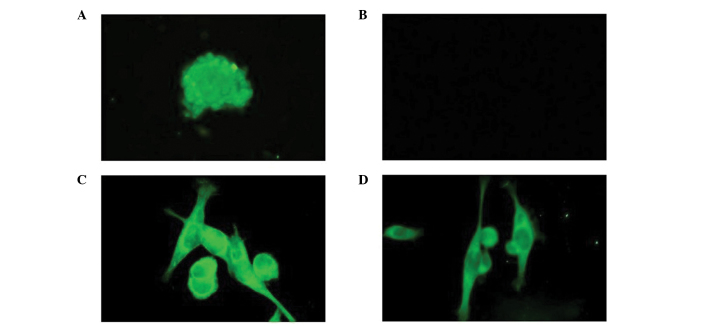
(A) Stem cell spheres were shown to stain positively for nestin (magnification, x200). (B) Stem cell spheres negative control (magnification, x200). (C) Stem cell spheres were induced for seven days in serum medium and were shown to stain positively for glial fibrillary acidic protein (magnification, x200). (D) Stem cell spheres were induced for seven days in serum medium and were shown to stain positively for β-tubulin (magnification, x200).

Following isolation using a magnetic-activated cell sorting technique, a small number of CD133^+^ stem cells were identified in the U251 cells. The stem cells started to differentiate following culture in DMEM/F12 containing 10% FBS. After culture for 4 h, the neurospheres adhered to the wall and axon-like or dendrite-like pseudopodia appeared. After four days of culture, the entire surface of the spheres emerged with pseudopodia. The stem cells cultured in the NSC medium did not exhibit similar changes. The stem cells that were cultured in NSC medium exhibited a sphere-like shape, self-renewal and the ability to differentiate. Nestin immunofluorescence staining of the stem cells was positive, whereas the U251 cells did not exhibit this feature. After the stem cells had been cultured for seven days in DMEM/F12, positive staining for β-tubulin and GFAP were observed, whereas the neurospheres did not exhibit this feature (Fig. 1).

#### Lentiviral transfection

**Figure 2.**
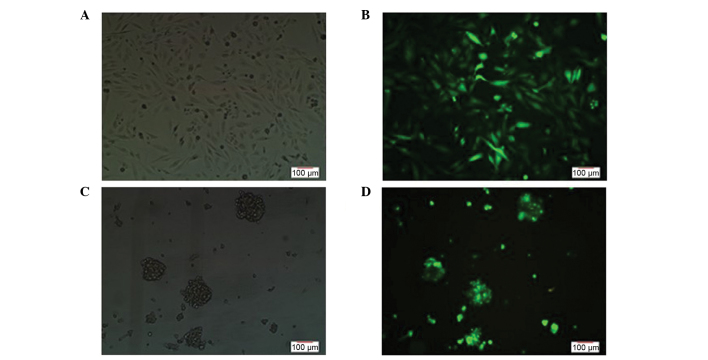
U251 cells (A) prior to and (B) following transfection. Stem cells (C) prior to and (D) following transfection.

Following transfection with the lentivirus for 72 h, the U251 cells and stem cells presented with green fluorescence under a confocal microscope (Fig. 2).

#### Proliferation analysis of U251 cells and stem cells using CCK-8

**Figure 3.**
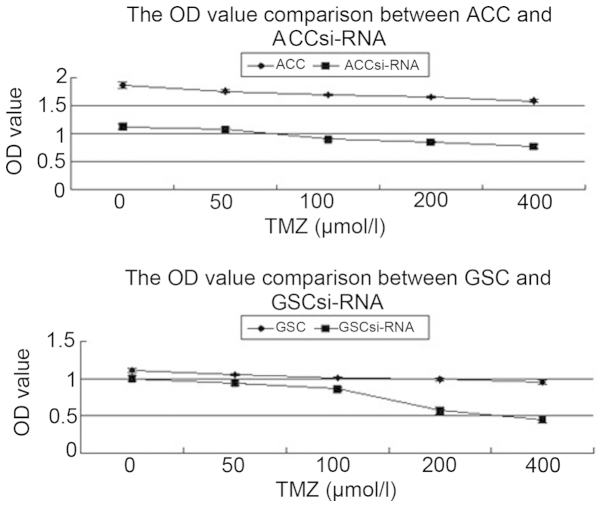
OD values of the four groups of cells following intervention with different concentrations of TMZ. TMZ, temozolomide; OD, optical density; GSC, glioma stem cell; ACC, autologous cancer cell.

Following intervention with TMZ for 24 h, the appreciation speed of the four types of cell was significantly inhibited. When compared with the autologous cancer cells (ACCs), the ACCs transfected with siRNA presented a lower proliferation rate (P<0.05). In addition, the GSCs transfected with siRNA presented a lower proliferation rate when compared with the GSCs (P<0.05; Fig. 3).

#### Morphological observations of the U251 cells and stem cells following TMZ intervention

**Figure 4.**
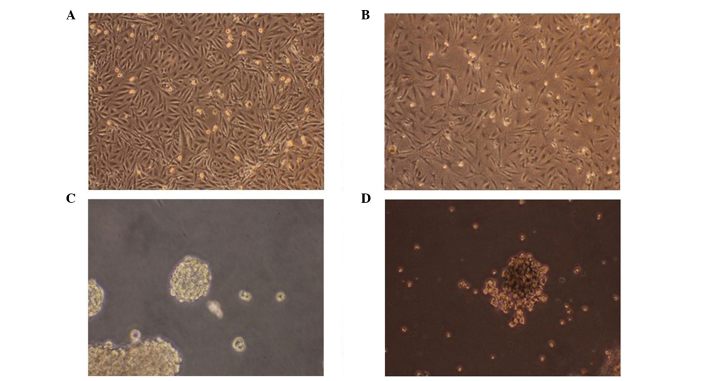
(A) U251 cells grew well with an intact cell structure and adherence to the bottom of the plate (magnification, x40). (B) With 400 µmol/l temozolomide (TMZ) intervention, the density of the cells reduced and cell debris was present, with the rate of cell proliferation significantly inhibited (magnification, x40). (C) U251 stem cells grew well in a sphere shape and were suspended in the medium (magnification, x40). (D) With 400 µmol/l TMZ intervention, the structure of the cell spheres was destroyed and a lot of cell debris was present, with cell proliferation significantly inhibited (magnification, x40).

Following intervention with TMZ, a decreased number of U251 cells were observed with cell fragmentation. The structures of the cell spheres were destroyed and a lot of cell debris was present, with the proliferation evidently inhibited. These changes were shown to be more significant with increasing drug concentrations and intervention times (Fig. 4).

#### Expression of Livin prior to and following transfection in U251 cells and stem cells

Prior to transfection, the expression of Livin in the U251 cells was 2.53±0.14×10^−5^, while following infection, the expression level was 4.74±0.47×10^−7^ (P<0.01). In the U251 stem cells prior to infection, the expression of Livin was 56.37±3.48×10^−5^, while following infection the expression level was 8.78±0.76×10^−7^ (P<0.01).

#### Expression of MRP1 and MRP3 prior to and following intervention with various concentrations of TMZ for 24 h

ACCs transfected with siRNA were shown to have higher mRNA expression levels of MRP1 when compared with the control ACCs (P<0.05). Under the same TMZ intervention time, the mRNA expression levels of MRP1 in the ACCs increased significantly with increasing concentrations of TMZ (P<0.05), while the expression levels decreased significantly in the ACCs transfected with siRNA (P<0.05). Following intervention with the same concentration of TMZ, the ACCs transfected with siRNA exhibited lower mRNA expression levels of MRP1 compared with those in the ACCs (P<0.05). The GSCs transfected with siRNA presented higher mRNA expression levels of MRP1 and MRP3 when compared with those in observed in the GSCs (P<0.05). In addition, the mRNA expression levels of MRP1 and MRP3 in the GSCs were shown to increase with increasing concentrations of TMZ (P<0.05). The expression of MRP3 in the GSCs transfected with siRNA increased with increasing concentrations of TMZ (P<0.05). However, the expression of MRP1 in the GSCs transfected with siRNA decreased significantly with increasing concentrations of TMZ (P<0.05). Following intervention with the same concentration of TMZ, the GSCs transfected with siRNA presented lower mRNA expression levels of MRP1 (P<0.05) and higher mRNA expression levels of MRP3 when compared with the control GSCs (P<0.05). Furthermore, following intervention with TMZ, the ACCs and GSCs transfected with siRNA presented a decreasing trend in MRP1 mRNA expression levels with increasing concentrations of TMZ. However, the GSCs transfected with siRNA presented an increasing trend in MRP3 mRNA expression levels with increasing concentrations of TMZ (Table I).

#### Expression of MRP1 and MRP3 following intervention with 400 µmol/l TMZ for 24, 48 and 72 h

The mRNA expression levels of MRP1 and MRP3 in the ACCs and GSCs presented increasing trends with increasing intervention times. Following intervention for the same time period, the mRNA expression levels of MRP1 in the ACCs transfected with siRNA were lower compared with those in the control ACCs (P<0.05). In addition, the mRNA expression levels of MRP1 in the GSCs transfected with siRNA were lower compared with those in the ACCs (P<0.05), while the mRNA expression levels of MRP3 in the GSCs transfected with siRNA were higher compared with those in the GSCs (P<0.05; Table II).

## Discussion

Glioblastoma is one of the most severe types of brain tumor. To date, a successful curative treatment has not been established using various therapies, including surgery, radiotherapy and chemotherapy. Although surgery has achieved great progress in recent years, including the use of intraoperative nuclear magnetic resonance, a curative outcome is yet to be achieved. Chemotherapy is widely used as an adjuvant treatment of gliomas following surgery, and TMZ is a commonly used chemotherapeutic for the treatment of primary and recurrent high-grade gliomas. The cytotoxic effect is the main antitumor mechanism underlying TMZ (13,14). Targeting DNA methylation, TMZ induces the rupture of double-stranded DNA, which subsequently interferes with DNA synthesis, induces autophagy and the apoptosis of tumor cells (15). TMZ has manifested a very strong lethality on glioma cells. However, the therapeutic outcome of TMZ is often unsatisfactory due to the antiapoptotic effect and the occurrence of chemotherapeutic resistance in glioma cells, particularly for GSCs. Overexpression of the antiapoptotic gene, Livin, results in a strong antiapoptotic effect in glioma cells and GSCs (16). There are a number of factors that can cause chemotherapeutic resistance in glioma, including MRP genes. The expression levels of MRP1 and MRP3 have been shown to increase with increasing concentrations of chemotherapeutic drugs in glioma cells and GSCs (16). Thus, targeted research of antiapoptotic genes and MRP genes may provide a novel direction for the treatment of glioblastoma.

Livin (also known as KIAP or ML-IAP) is a member of apoptosis protein suppressor family. The protein plays a key role in cell apoptosis, cell proliferation and the cell cycle. The expression of Livin is significantly higher in U251 stem cells when compared with U251 glioma cells (16). Overexpression of Livin can lead to the chemotherapeutic resistance of malignant cells; thus, inhibitors of Livin may be useful for the chemotherapy of malignancies (17).

MRPs, one of the most important causes of poor prognosis in cancer patients, belong to the ATP-associated transporter family. The proteins have a molecular weight of 180–190 kDa and are associated with the transfer of hydrophobic compounds. MRP1 and MRP3 are the two most important genes in the family. MRP1 has an unusually broad substrate specificity that can transport a variety of neutral hydrophobic compounds and facilitate the extrusion of numerous glutathione, glucuronate and sulfate conjugates (18). However, the transport mechanism of MRP1 is unknown. MRP1 is able to confer drug resistance *in vitro*, and MRP1 has been hypothesized to play an important role in the development of drug resistance in several types of cancer (19). MRP3 is able to transport organic compounds that are conjugated to glutathione, sulfate or glucuronate, such as estradiol-17β-glucuronide, bilirubin-glucuronides and etoposide-glucuronide, as well as bile salts and methotrexate (20). To date, there have been a limited number of studies investigating the associations between MRPs and glioblastoma.

In a previous study, the difference in the mRNA expression levels of MRP1 and MRP3 between glioma cells and GSCs was confirmed. Compared with glioma cells, the expression of MRP1 is significantly increased, while MRP3 expression is significantly decreased in GSCs (10). The antiapoptotic gene, Livin, has been shown to be associated with MRP1 and MRP3 (16). Thus, the present study assessed Livin as a target to investigate the association between Livin and MRP. Identifying an approach that can reduce the chemotherapy resistance of glioma cells and GSCs may become a novel therapeutic method for glioma cases. Thus, the present study investigated the effect of siRNA-Livin on the expression of MRP1 and MRP3.

In the present study, the effect of the antiapoptotic gene, Livin, on the expression of MRP1 and MRP3 was investigated following chemotherapy in glioma cells and GSCs. The results demonstrated that following lentiviral transfection of siRNA-Livin, the expression of Livin was significantly inhibited in U251 cells and GSCs. Prior to siRNA-Livin transfection, the expression levels of MRP1 increased in the U251 and stem cells with increasing drug concentrations and intervention times. However, following siRNA-Livin transfection, the expression of MRP1 was shown to decrease in U251 cells and GSCs under the same drug concentration and intervention time. By contrast, the expression of MRP3 increased in the U251 stem cells under the same intervention concentration and time.

In conclusion, siRNA-Livin was shown to decrease the expression levels of MRP1 in U251 cells and U251 stem cells, while increasing the expression levels of MRP3 in U251 stem cells. Furthermore, siRNA-Livin decreased the proliferation of U251 cells and GSCs. Livin may be associated with high expression levels of MRP1; thus, siRNA-Livin may decrease the expression of MRP1 to subsequently reduce the drug resistance to chemotherapy in glioblastoma. However, the effect of siRNA-Livin on MRP3 expression in CD133^+^ stem cells requires further study.

## Figures and Tables

**Table I.** mRNA expression levels of MRP1 and MRP3 prior to and following TMZ intervention for 24 h.

Cell type	No intervention	50 µmol/l	100 µmol/l	200 µmol/l	400 µmol/l
MRP1 (x10^−4^)					
ACC	4.570±0.410	6.161±0.693^c^	6.688±0.809^c^	7.232±0.820^d^	7.848±1.006^d^
ACC siRNA	6.315±0.522^a^	4.678±0.240^d,a^	4.354±0.438^d,a^	4.094±0.187^d,b^	2.703±0.182^d,b^
GSC	5.527±0.528	7.287±0.902^c^	8.058±0.765^d^	8.818±0.977^d^	13.108±1.478^d^
GSC siRNA	9.074±0.746^b^	6.861±0.640^c^	4.351±0.472^d,b^	3.610±0.269^d,b^	3.399±0.297^d,b^
MRP3 (x10^−4^)					
ACC	25.419±4.272	31.101±3.002	27.615±3.946	17.894±1.963^c^	14.365±1.789^c^
ACC siRNA	23.384±2.879	25.819±2.834	22.650±1.424	19.781±1.840	17.631±1.798^c^
GSC	7.614±0.748	9.605±0.722^c^	10.798±1.350^c^	12.616±1.084^d^	17.464±1.658^d^
GSC siRNA	19.537±0.923^b^	15.704±1.322^c,b^	21.469±1.949^b^	24.638±1.056^d,b^	28.448±2.504^d,b^

Results are expressed as the mean ± standard deviation.

a P<0.05

b P<0.01, vs. control group of the same cells at the same concentration

c P<0.05

d P<0.01, vs. same cells without any intervention. ACC, autologous cancer cell; GSC, glioma stem cell; MRP, multidrug resistance-associated protein; TMZ, temozolomide.

**Table II.** mRNA expression levels of MRP1 and MRP3 prior to and following 400 µmol/l TMZ intervention.

Cell type	No intervention	24 h	48 h	72 h
MRP1 (x10^−4^)				
ACC	4.570±0.410	7.848±1.006^d^	13.033±1.560^d^	19.323±3.056^d^
ACC siRNA	6.315±0.522^a^	2.703±0.182^d,b^	4.275±0.622^c,b^	3.849±0.411^d,b^
GSC	5.527±0.528	13.108±1.478^d^	12.613±1.182^d^	27.372±2.062^d^
GSC siRNA	9.074±0.746^b^	3.399±0.297^d,b^	3.855±0.658^d,b^	5.791±0.585^d,b^
MRP3 (x10^−4^)				
ACC	25.419±4.272	14.365±1.789^c^	29.846±5.499	32.852±5.154
ACC siRNA	23.384±2.879	17.631±1.798^c^	23.676±3.848	32.646±3.815^c^
GSC	7.614±0.748	17.464±1.658^d^	18.196±1.961^d^	27.197±1.963^d^
GSC siRNA	19.537±0.923^b^	28.448±2.504^d,b^	33.092±3.872^d,b^	45.918±4.146^d,b^

Results are expressed as the mean ± standard deviation.

a P<0.05

b P<0.01, vs. control group of the same cells at the same concentration

c P<0.05

d P<0.01, vs. same cells without any intervention. ACC, autologous cancer cell; GSC, glioma stem cell; MRP, multidrug resistance-associated protein; TMZ, temozolomide.

## Abbreviations

MRP: multidrug resistance-associated protein
GSC: glioma stem cell
TMZ: temozolomide
FBS: fetal bovine serum
EGF: epidermal growth factor
bFGF: basic fibroblast growth factor
LIF: leukemia inhibitory factor
DMEM/F12: Dulbecco's modified Eagle's medium/nutrient mixture F-12 Ham's
CCK-8: Cell Counting Kit-8
ACC: autologous cancer cell
NSC: neural stem cell
PCR: polymerase chain reaction
